# Characterization of the inheritance of field-evolved resistance to diamides in the fall armyworm (*Spodoptera frugiperda*) (Lepidoptera: Noctuidae) population from Puerto Rico

**DOI:** 10.1371/journal.pone.0295928

**Published:** 2024-02-23

**Authors:** Omar Posos-Parra, David Mota-Sanchez, Barry R. Pittendrigh, John C. Wise, Christina D. DiFonzo, Eric Patterson

**Affiliations:** 1 Department of Entomology, Michigan State University, East Lansing, Michigan, United States of America; 2 Department of Entomology, Purdue University, West Lafayette, Indiana, United States of America; 3 Department of Plant, Soil, and Microbial Sciences, Michigan State University, East Lansing, Michigan, United States of America; King Saud University, SAUDI ARABIA

## Abstract

The fall armyworm (*Spodoptera frugiperda*) is one of the most destructive pests of corn. New infestations have been reported in the East Hemisphere, reaching India, China, Malaysia, and Australia, causing severe destruction to corn and other crops. In Puerto Rico, practical resistance to different mode of action compounds has been reported in cornfields. In this study, we characterized the inheritance of resistance to chlorantraniliprole and flubendiamide and identified the possible cross-resistance to cyantraniliprole and cyclaniliprole. The Puerto Rican (PR) strain showed high levels of resistance to flubendiamide (RR_50_ = 2,762-fold) and chlorantraniliprole (RR_50_ = 96-fold). The inheritance of resistance showed an autosomal inheritance for chlorantraniliprole and an X-linked inheritance for flubendiamide. The trend of the dominance of resistance demonstrated an incompletely recessive trait for H1 (♂ SUS × ♀ PR) × and an incompletely dominant trait for H2 (♀ SUS × ♂ PR) × for flubendiamide and chlorantraniliprole. The PR strain showed no significant presence of detoxification enzymes (using synergists: PBO, DEF, DEM, and VER) to chlorantraniliprole; however, for flubendiamide the SR = 2.7 (DEM), SR = 3.2 (DEF) and SR = 7.6 (VER) indicated the role of esterases, glutathione S- transferases and ABC transporters in the metabolism of flubendiamide. The PR strain showed high and low cross-resistance to cyantraniliprole (74-fold) and cyclaniliprole (11-fold), respectively. Incomplete recessiveness might lead to the survival of heterozygous individuals when the decay of diamide residue occurs in plant tissues. These results highlight the importance of adopting diverse pest management strategies, including insecticide rotating to manage FAW populations in Puerto Rico and other continents.

## Introduction

The fall armyworm (FAW), *Spodoptera frugiperda* (J.E. Smith) (Lepidoptera: Noctuidae), is one of the most destructive pests of corn. It is native to the Americas, mainly Latin America, Caribbean islands, and the extreme southern part of the US, with annual migrations north into the US corn belt and Canada [1, 2]. In 2016, infestations were found for the first time in Africa, setting the stage for a dramatic change in its distribution and economic impacts [3, 4]. Its current expanded range includes India, China, Japan, Malaysia, Vietnam, Egypt, the Republic of Korea, and Australia [5–9]. FAW has several traits that make it one of the most economically important pests of the 21^st^ century including a high reproductive rate, no diapause, and the ability to rapidly adapt to new environments, including the adaptation to novel insecticides [10, 11]. There are 192 reported cases of FAW resistance to 43 different active ingredients belonging to eight modes of action [12].

In addition to direct losses to food and feed from FAW, losses in the seed industry are often overlooked. Puerto Rico plays a crucial role in agricultural seed production for both research and bulk seed production. Its tropical climate allows for three to four seasons of corn production per year. It is estimated that 85% of all certified field crop seeds used for food consumption worldwide pass-through Puerto Rico’s fields and nurseries at some point in development [13]. However, tropical conditions are optimal for FAW populations, resulting in high and constant pest pressure in seed corn fields. To manage FAW injury, there is high pesticide usage, with up to 30 applications per season of products in at least nine modes of action [14]. As a result of such constant pest pressure and insecticide use, FAW populations in Puerto Rico have developed resistance to a wide diversity of synthetic insecticides [15] and to *Bacillus thuringiensis* (Bt) proteins in GMO corn [16–21].

With the increase in FAW resistance globally, diamide insecticides (Group 28, IRAC) have become one of the critical tools for integrated pest management (IPM) of this species. The diamide insecticides selectively activate ryanodine receptors (RyR) in the endoplasmic reticulum of insects, a novel mode of action in Lepidoptera pests [6, 22]. RyR modulate the release of Ca^2+^ cations from intracellular stores, allowing insect muscles to contract. When diamides activate RyRs, insects suffer irreversible muscle contraction and paralysis [22–26]. They are highly selective against insects and exhibit reduced toxicity in mammals. In general, they are safer and more ecologically friendly than some older insecticides. For instance, the acute oral toxicity of chlorantraniliprole is much lower than that of chlorpyrifos, with LD50 values of over 5,000 mg/kg (in rats) and over 60 mg/kg (in rats), respectively [27, 28]. As a result, diamides use to be one of the most common MOAs used today [6, 29]. Diamide insecticides primarily target Lepidopteran species which is very important since many Lepidopteran species have developed extensive resistance to pesticides. Additionally, diamides are also effective against other order as Coleoptera and Hemiptera [12, 30].

The diamides, chlorantraniliprole and flubendiamide, have both been heavily used to control FAW in seed corn production in Puerto Rico since its registration in 2016 [31]. Flubendiamide is not used anymore due to environmental restrictions [32]; however, chlorantraniliprole continues to be used. Resistance to these compounds was recently detected in FAW populations from Puerto Rico [15]. However, the inheritance and mechanism of this resistance remains unknown. Understanding these aspects is essential for managing resistance and prolonging the use of diamide products.

The objective of this study was to determine the inheritance and begin to explore the mechanisms of resistance using synergists to diamide insecticides in Puerto Rican FAW from seed corn production. This work increases our understanding of the inheritance of this resistance and its mechanism, as well as provides insights into FAW management in Puerto Rico and globally.

## Materials and methods

### FAW strains

An (assumed) diamide-resistant strain [PR] originated directly collected from infested plants in a seed corn field in Ponce, Puerto Rico, in January 2019. Larvae were collected by personnel of Corteva under a collaboration Michigan State University-Corteva. Larvae were placed in 60 mL plastic cups with 5 mL of artificial FAW diet (Southland Products Inc., Lake Village, AR) and shipped to our laboratory at Michigan State University, East Lansing, MI to complete development under controlled conditions. Larvae were checked every three days to monitor feeding and development. The known diamide-susceptible population [SUS] was provided by Bayer USA from their rearing facilities in Union City, Tennessee. We have been using this susceptible strain for at least 8 years.

### Colony maintenance

Larvae were maintained under controlled conditions (26 ± 2 ºC; 35 ± 2% RH) and a photoperiod of 16:8 hours (L:D), inside an FXC-19 Growth Chamber (BioChamber, Winnipeg, Manitoba, Canada) After pupation, twenty pairs of pupas (even number male and female) were placed in a 5 L paper brown bag to provide space for mating and egg-laying. Bags were placed inside of cylindric mesh cages to avoid accidental escape. Adults were fed lime or orange liquid Gatorade (PepsiCo, Harrison NY). Egg masses were collected every other day by cutting out the sections of the bag containing the egg masses. These were collected every other day and placed in a closed plastic container with moist paper towels. Egg masses were transferred to 60 mL plastic cups with 10 mL of artificial FAW diet to ensure safe emergence and efficient feeding from the beginning of the larval cycle. As eggs hatched, multiple neonates were moved to diet cups using a fine paintbrush. Once reaching third instar stage, larvae were collected either bioassays or for the colony, placing one larva per cup.

### Diet overlay bioassays—General methods

Diet overlay bioassays were performed using 24-well trays (ProCell, Alkali Scientific Inc., Fort Lauderdale, FL) with 1 mL of artificial FAW diet per well. The diet surface area in each well was 2.0 cm^2^. Products to be tested and appropriate controls were applied to the diet and left to air dry for an hour. A total of 30 μL of insecticide or control solution was applied to each well. Then one early third instar was deposited on the treated surface of each well. Mortality was recorded four days after placing the larva over the treated diet. Larvae were considered dead if they did not react after prodding insect with a small paintbrush or showed severe intoxication symptoms (defined as slow movement, interrupted molting, or reduced size). We selected the overlay diet assay over diet [33, 34] incorporated assay because the fast and clean procedure to perform this type of bioassays.

Mortality data was corrected using Abbott’s equation [35]. Probit analysis [36] was performed using the PROC PROBIT procedure from SAS version 9.4 [37] to estimate slope values, median lethal concentration 50% (LC_50_), and 90% (LC_90_), fiducial limits (95%), and X^2^ for each strain. Resistance ratios at 50% and 90% (RR_50_ and RR_90_) were calculated by dividing LC_50_ or LC_90_ values of the PR strain by the LC_50_ or LC_90_ values of the SUS strain. Where appropriate, parallelism and equality tests (*P<0*.*05*) were also calculated to compare the responses of the strains to the diamide compounds using the software PoloJR [38]. Log concentration responses were plotted using the software OriginLab [39].

### Evaluating resistance to diamides used in PR seed corn

For both the PR and SUS populations, bioassays were done for two diamide formulations: chlorantraniliprole (Altacor^®^ 35 WG, 35 g a.i./kg, FMC Corporation, Philadelphia, PA) and flubendiamide (Belt^®^ 480 SC, 480 g a.i./L, Bayer CropScience LP, Research Triangle Park, NC). The formulated material was resuspended in distilled water, and non-ionic surfactant (Triton X-100, Sigma-Aldrich, Merck KGaA, Darmstadt, Germany) was added in a concentration of 0.05% v/v. Each insecticide was tested at concentrations covering a range of mortality from 5% to 95% (S1 Table). Four to five replications per concentration were performed. A single replication consisted of twelve wells with one early third-instar per well. The control treatment consisted of distilled water with only the surfactant. Mortality was assessed at four days, as described in the general methods.

Bioassays were performed using the procedure for both strains to determine if there was cross-resistance among diamides. Diamides tested were cyclaniliprole (Harvanta^®^ 50 SL, Summit Agro USA, Durham, NC) and cyantraniliprole (Exirel^®^ 100 SE, FMC Corporation, Philadelphia, PA); both insecticides are not used in cornfields in PR. However, they are used in several countries against a wide range of pest, including lepidopteran pests, beetles, and various types of flies and bugs, in crops such as citrus, vegetables, and rice [40]. Thus, they are good candidates to understand the cross-resistance in FAW among diamides compounds.

### Determining inheritance of resistance

F_1_ crosses between the two FAW populations (PR and SUS) were tested to determine the inheritance of resistance to chlorantraniliprole and flubendiamide. Forty reciprocal pairs of FAW were separated to create the F1 crosses in heterozygous (H) populations, creating two F1 crosses defined as H1 = ♂ SUS × ♀ PR) and H2 = (♀ SUS × ♂ PR), which were bioassayed in the same way as the parental strains. Four replicates were use in seven to nine concentrations (S1 Table) to cover a range of mortality from 5% to 95%.

The degree of dominance at the LC_50_ level was calculated as follows using Stone’s equation [41],

$$D=\frac{2X_{2}-X_{1}-X_{3}}{X_{1}-X_{3}}$$ (1)

where X_2_, X_1_, and X_3_ were the log10 LC_50_ of the F_1_ (H1 or H2 strain), PR strain, and SUS strains. When *D* values = -1 signify complete recessive, *D* values = -1 < *D < 0* signify incomplete recessive, *D* values = 0 < *D <* 1 signify incomplete dominant and *D* = 1 signify complete dominant.

Dominance level (*D*_*ML*_) using concentrations that span the log concentration mortalities was calculated using the method described by Bourguet, *et al*. [42],

$$D_{ML}=\frac{M_{RS}-M_{SS}}{M_{RR}-M_{SS}}$$ (2)

where M_SS_, M_RS_, and M_RR_ were the mortalities of the SUS, F_1_ (H1 or H2 strain), and PR strains, respectively, at different concentrations of each diamide. *D*_*ML*_ values close to 0 were considered completely-recessive inheritance, *D*_*ML*_ values approaching 1—completely-dominant inheritance. This method considered testing a range of concentrations covering the parental and F_1_ crosses log concentration responses to understand the trend of dominance/recessiveness.

### Role of detoxification enzymes

To determine the role of detoxification enzyme in the resistance of diamides, synergists bioassays were performed as the following: (i) a cytochrome P450s inhibitor, piperonyl butoxide (PBO) (91.3%, SynerPro^™^ Control Solutions Inc. Pasadena, Texas, USA); (ii) an esterase inhibitor, S,S,S-tributyl phosphorotrithioate (DEF) (98.1%, Sigma-Aldrich, Saint Louis, Missouri, USA); (iii) a glutathione S-transferase inhibitor, diethyl maleate (DEM) (97%, Sigma-Aldrich, Saint Louis, Missouri, USA); (iv) an ABC transporters inhibitor (±)-verapamil hydrochloride (VER) (99%, Sigma-Aldrich, Saint Louis, Missouri, USA). Control treatment consisted of synergist application over the diet without pesticide. Prior to the bioassay with the diamides, the maximum non-lethal concentration of each synergist was assessed on third instars using the diet overlay bioassay method. The criteria to identify the maximum nonlethal concentration of each synergist was that which did not account for significantly higher mortality or loss of fitness (weight) in the larva four days after application compared to water control. The maximum non-lethal concentrations determined for PBO, DEF, DEM and VER per diet surface were 4.5 μg/cm^2^, 1.5 μg/cm^2^, 0.45 μg/cm^2^ and 0.45 μg/cm^2^, respectively.

Using the maximum non-lethal concentrations, synergists bioassays were performed. Stock solutions of synergist compounds were prepared by diluting in distilled water. A non-ionic surfactant (Triton X-100, Sigma-Aldrich, Merck KGaA, Darmstadt, Germany) was added in a concentration of 0.05%. A total of 30 μL of synergist was applied over the diet surface of each well and left to air dry for 1 hour. Then, 30 μL of each insecticide concentration was applied over the same surface well and left to air dry. Finally, one third instar was placed in each well treated. Mortality was assessed four days after application. The experimental design consisted of four replicates of five to seven concentrations, and a single replication consisted of twelve wells with one third instar per well.

Scoring of mortality probit analyses and data plotting were estimated following the procedure mentioned before. Synergist ratios (SR_50_ and SR_90_) were calculated by dividing LC_50_ and LC_90_ values of the diamide alone by the LC_50_ and LC_90_ values of the diamide plus synergist combination.

## Results

### Inheritance of resistance

**Fig 1:**
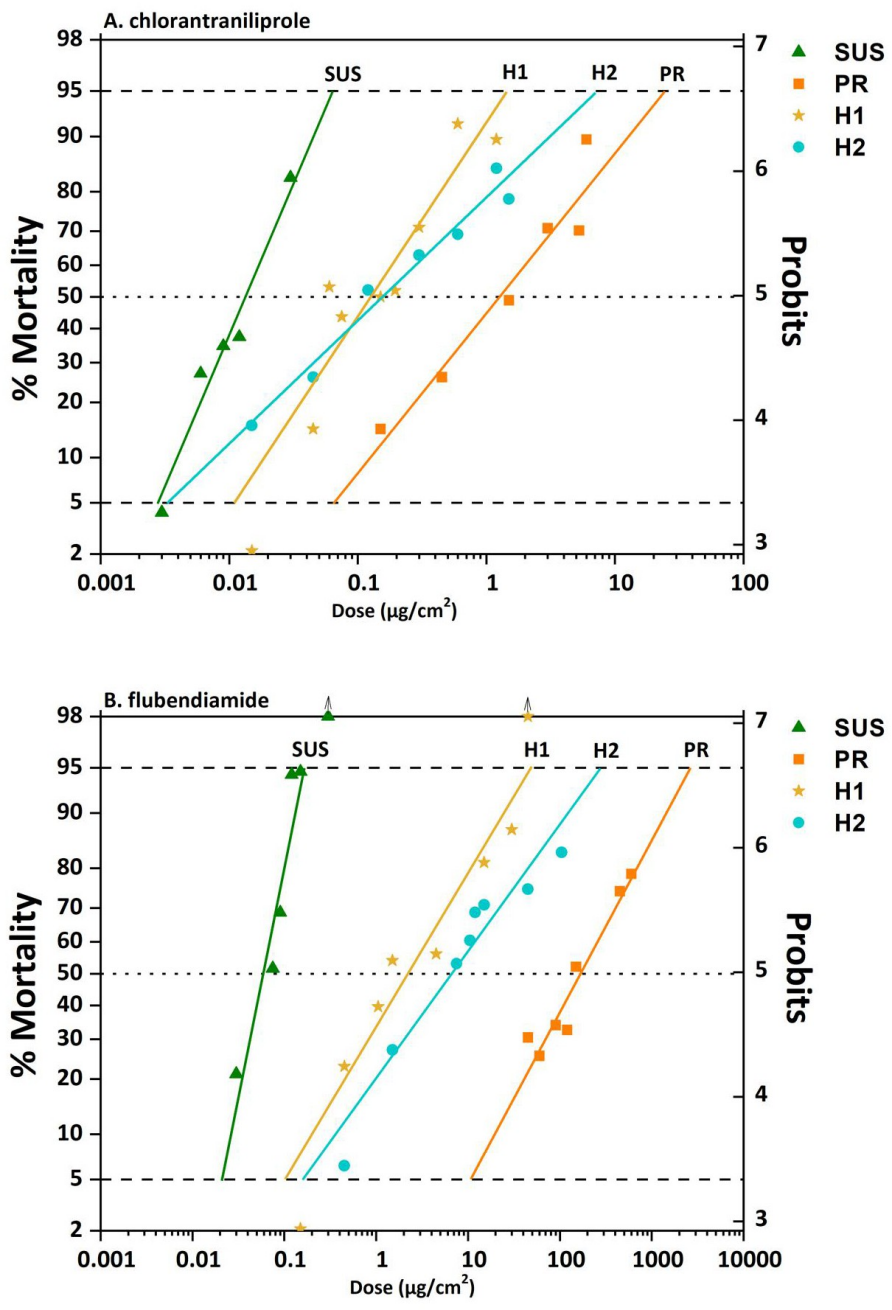
Mortality of a susceptible (SUS) and resistant (PR) strain and their reciprocal crosses (H1, ♂ SUS × ♀ PR) + (H2, ♀ SUS × ♂ PR) to A. chlorantraniliprole and B. flubendiamide.

The PR strain showed high levels of resistance to flubendiamide and chlorantraniliprole, RR_50_ = 2,762-fold, and RR_50_ = 96-fold over the susceptible, respectively. The F_1_ progenies from reciprocal crosses (H1♀ PR × ♂ SUS and H2 ♂ PR × ♀ SUS) presented similar susceptibility to chlorantraniliprole with overlapping confidence levels for the LC_50_ (95% CI) 0.126 (0.07, 0.19) and 0.155 (0.10, 0.22). In contrast, susceptibility of F_1_ progenies from reciprocal crosses (H1♀ PR × ♂ SUS and H2 ♂ PR × ♀ SUS) to flubendiamide were different with LC_50_ of 2 (1.2, 3.5) and 7 (4.5, 8.9), respectively (Table 2). Equality tests for chlorantraniliprole (X^2^ = 14.63, d.f. = 4, *P<0*.*05*) and flubendiamide (X^2^ = 95.93, d.f. = 4, *P<0*.*05*) demonstrated that the heterozygote strains were different to each other. Compared to the SUS strain, resistance ratios (RR_50_) for H1 and H2 were 10-fold and 12-fold for chlorantraniliprole and 37-fold and 111-fold for flubendiamide, respectively (Table 1). The overlapping of the LC_50_ between confidence intervals (95% CI) of H1 and H2 strains in chlorantraniliprole suggested an autosomal inheritance of resistance. For flubendiamide the results suggested an X-linked inheritance of resistance (Fig 1).

**Table 1:** Dose-response of a resistant (PR) strain and a susceptible (SUS) of FAW and their reciprocal crosses to chlorantraniliprole and flubendiamide.

Strain	active ingredient	n	^e^b	SE	^a^LC_50_ (95% CI)	^a^LC_90_ (95% CI)	^b^RR_50_	^b^RR_90_	X^2^	^c^df	^d^ *D*
PR	chlorantraniliprole	284	1.3	0.2	1.26 (0.92, 1.69)	12.82 (7, 26)	96	287	5.4	4	
SUS		236	2.4	0.3	0.013 (0.11, 0.016)	0.045 (0.03, 0.08)	1	1	3.2	3	
H2 ♀ SUS × ♂ PR		332	1.0	0.1	0.155 (0.10, 0.22)	3.072 (1.7, 7.3)	12	69	2.6	5	0.079
H1 ♂ SUS × ♀ PR		427	1.5	0.3	0.126 (0.07, 0.19)	0.857 (0.4, 3.2)	10	19	21.6	7	-0.011
PR	flubendiamide	333	1.4	0.2	162.42 (128, 210)	1,375 (808, 3,359)	2,762	10,316	4.8	5	
SUS		304	3.6	0.4	0.0588 (0.04, 0.06)	0.13 (0.11, 0.16)	1	1	8.5	5	
H2 ♀ SUS × ♂ PR		383	1.0	0.1	6.51 (4.5, 8.9)	122.44 (71, 271)	111	919	6.2	6	0.018
H1♂ SUS × ♀ PR		384	1.2	0.2	2.149 (1.2, 3.5)	24.04 (12, 74)	37	180	11.7	6	-0.092

^a^ LC_50_ or LC_90_ (μg/cm^2^)

^b^ Resistance ratio (RR), LC_50_ of resistant strain / LC_50_ of susceptible strain or LC_90_ of resistant strain / LC_90_ of susceptible strain

^c^ df = degrees of freedom

* chlorantraniliprole (Altacor^®^ 35 WG, 35 g a.i./kg, FMC Corporation, Philadelphia, PA)

* flubendiamide (Belt^®^ 480 SC, 480 g a.i./L, Bayer CropScience LP, Research Triangle Park, NC)

^d^ degree of dominance using Stone’s equation (1968)

^e^ slope

### Dominance of resistance

**Fig 2:**
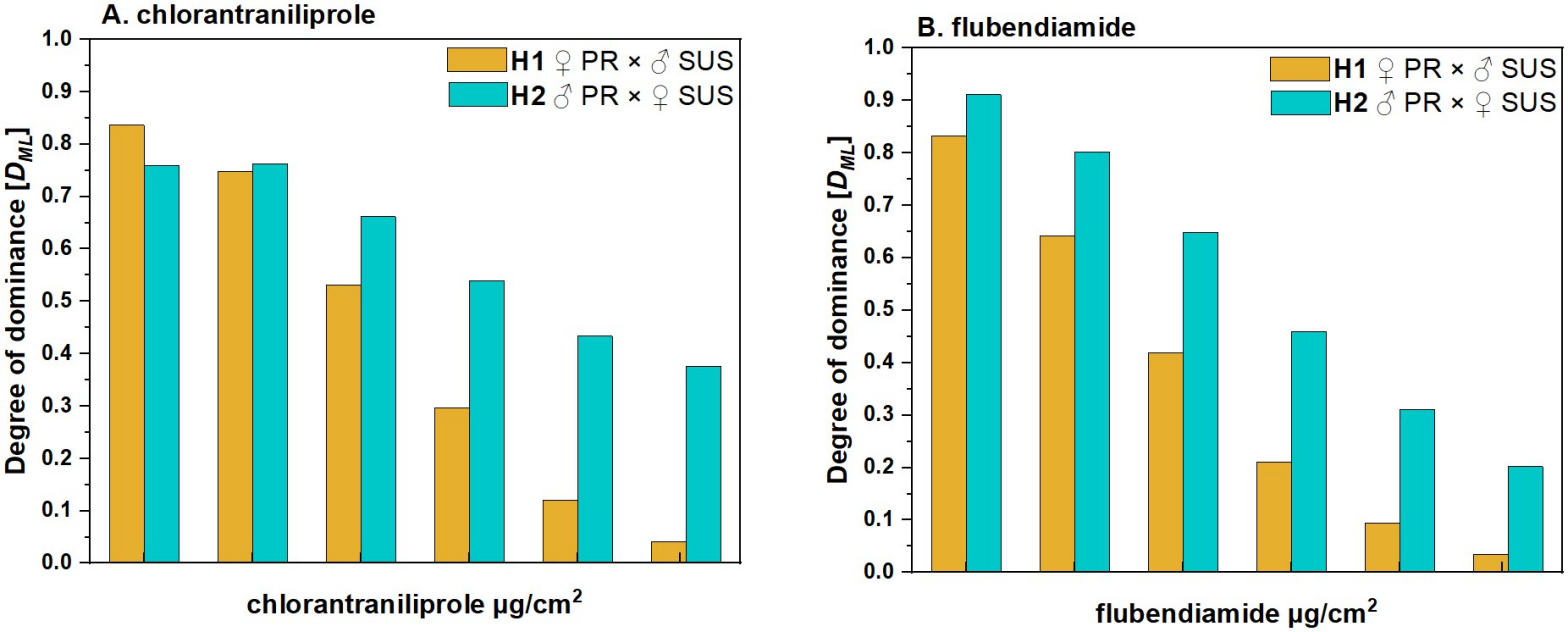
Degree of dominance (*D*_*ML*_) of resistance of FAW to chlorantraniliprole and flubendiamide.

The degree of dominance (*D*) at LC_50_ was -0.011 and 0.079 for chlorantraniliprole and -0.092 and 0.018 for flubendiamide using the method proposed by Stone [41], suggesting incompletely recessive trait for H1 strain (♂ SUS × ♀ PR) and incompletely dominant trait for H2 strain (♀ SUS × ♂ PR), in both chlorantraniliprole and flubendiamide, respectively. For both active ingredients, dominance decreased indirectly proportional to the concentration. At low doses of active ingredients, dominance is incompletely dominant, while at high doses, is incompletely recessive (Fig 2). For instance: at the low concentration tested (0.01 μg/cm^2^ of chlorantraniliprole) showed a value *D*_*ML*_*<0*.*83*, whereas (0.3 μg/cm^2^ of flubendiamide) showed a value of *D*_*ML*_*<0*.*92*. At the highest concentration tested (3 μg/cm^2^ of chlorantraniliprole) showed a value of *D*_*ML*_*<0*.*41*, whereas (100 μg/cm^2^ of flubendiamide) showed a value of *D*_*ML*_*<0*.*20* (Fig 2).

### Role of detoxification enzymes

In the chlorantraniliprole-synergist bioassays, the LC_50_, LC_90_ values with or without exposure to PBO, DEF, DEM, and VER did not differ significantly based on the overlap between the LC_50_ confidence intervals (95% CI) in each PR and SUS strains (Table 2). However, there was a slight increase in toxicity with the esterase inhibitor (DEF) and the glutathione S-transferase inhibitor (DEM) in the PR strain of chlorantraniliprole (2-fold) compared to the insecticide without synergist.

**Table 2:** Effect of synergists on mortality of Puerto Rico’s (PR) and Susceptible (SUS) strains of FAW to chlorantraniliprole.

Strain	Synergists	N	^d^b	SE	^a^LC_50_ (95% CI)	^a^LC_90_ (95% CI)	^b^SR_50_	^b^SR_90_	X^*2*^	^c^df
PR	chlorantraniliprole	284	1.3	0.2	1.2659 (0.92, 1.69)	12.82 (7, 26)	-	-	5.4	4
	+ PBO	287	1.7	0.2	1.461 (1.13, 1.83)	8.16 (5.8, 13)	1	1	6	4
	+ DEM	236	0.9	0.2	1.19 (0.69, 1.8)	33.99 (13, 260)	1.2	0.2	3.8	3
	+ DEF	307	1.2	0.2	1.49 (0.62, 2.6)	19.39 (8, 116)	1	0.4	10	5
	+ VER	288	1.0	0.1	1.043 (0.7, 1.47)	17.84 (9, 48)	1.4	0.5	7	4
SUS	chlorantraniliprole	236	2.4	0.3	0.0132 (0.11, 0.016)	0.045 (0.03, 0.08)	-	-	3.2	3
	+ PBO	240	2.1	0.3	0.0150 (0.012, 0.019)	0.06 (0.04, 0.12)	1	1	5.4	3
	+ DEM	336	1.5	0.3	0.0078 (0.004, 0.013)	0.056 (0.026, 0.34)	2	1.1	14	5
	+ DEF	240	2.0	0.2	0.0073 (0.005, 0.009)	0.032 (0.023, 0.051)	2	2	2	3
	+ VER	336	1.9	0.3	0.0135 (0.007, 0.021)	0.064 (0.038, 0.15)	1.1	1	13	5

^a^ LC_50_ or LC_90_ (μg/cm^2^)

^b^ Synergist ratio (SR) = LC_50_ of chlorantraniliprole without synergist / LC_50_ of chlorantraniliprole + synergist.

^c^ df = degrees of freedom

PBO = piperonyl butoxide, DEM = diethyl maleate, DEF = S,S,S -tributyl phosphorotrithioate and VER = (±)-verapamil hydrochloride

^d^ slope

In the flubendiamide bioassays, the LC_50_’s values demonstrated a different response in both the SUS and PR strains. A minor antagonism (<1-fold) was found in the SUS strain with all the synergists. While, in the PR strain, there was no synergism (0.9-fold) with the P450s inhibitor (PBO), this was a case not the case with the other synergists. For instance, the esterase inhibitor (DEF) and the glutathione S-transferase inhibitor (DEM) showed moderate synergism with 2.7-fold and 3.2-fold, respectively, and the ABC transporters inhibitor (VER) demonstrated a moderate-high synergism with 7.6-fold at the LC_50_ dosages. Yet, in all synergist combinations with flubendiamide, the synergism was low at the LC_90_ dosages (Table 3).

**Table 3:** Effect of synergists on mortality of Puerto Rico’s (PR) and Susceptible (SUS) strains of FAW to flubendiamide.

Strain	Synergists	n	^d^b	SE	^a^LC_50_ (95% CI)	^a^LC_90_ (95% CI)	^b^SR_50_	^b^SR_90_	X^*2*^	^c^df
PR	flubendiamide	333	1.4	0.2	162.42 (128, 210)	1,375 (808, 3,359)	-	-	4.8	5
	+ PBO	239	1.3	0.2	175.07 (126, 244)	1,715 (992, 4,006)	0.9	0.8	5	3
	+ DEM	239	1.1	0.1	60.04 (40, 89)	907 (482, 2,339)	2.7	1.5	5	3
	+ DEF	335	1.0	0.1	50.01 (24, 102)	1,095 (406, 6,981)	3.2	1.3	10	5
	+ VER	240	0.8	0.1	21.31 (11, 34)	795 (356, 3,130)	7.6	1.7	3	3
SUS	flubendiamide	304	3.6	0.4	0.059 (0.04, 0.06)	0.133 (0.11, 0.16)	-	-	8.5	5
	+ PBO	335	2.0	0.3	0.098 (0.06, 0.13)	0.44 (0.26, 1.40)	0.6	0.3	11	5
	+ DEM	288	1.3	0.5	0.144*	1.3614*	0.4	0.1	31	4
	+ DEF	238	1.7	0.2	0.141 (0.10, 0.17)	0.812 (0.53, 1.6)	0.4	0.2	4	3
	+ VER	285	3.9	0.7	0.161 (0.11, 0.21)	0.3 (0.25, 0.75)	0.4	0.4	11	4

^a^ LC_50_ or LC_90_ (μg/cm^2^)

^b^ Synergist ratio (SR) = LC_50_ of flubendiamide without synergist / LC_50_ of flubendiamide + synergist

^c^ df = degrees of freedom

* No confidence intervals could be calculated

PBO = piperonyl butoxide, DEM = diethyl maleate, DEF = S,S,S -tributyl phosphorotrithioate and VER = (±)-verapamil hydrochloride

^d^ slope

These results suggest that the metabolic resistance differs between chlorantraniliprole and flubendiamide. In the chlorantraniliprole synergist bioassays indicate that there is little evidence of metabolic resistance while the flubendiamide-synergism results indicate the presence of metabolic resistance in FAW strain from Puerto Rico (PR) at concentrations around the LC_50_.

### Cross-resistant among diamides

The Puerto Rico (PR) strain showed cross-resistance to all tested diamide insecticides, including cyantraniliprole (11-fold) and cyclaniliprole (74-fold) (Table 4). However, LD_50_s, and LC_90_s were not significant different in the PR strain between chlorantraniliprole and cyclaniliprole (Table 4). The equality (X^2^ = 45.48, d.f. = 7, *P<0*.*05*) and parallelism (X^2^ = 58.95, d.f. = 5, *P<0*.*05*) tests for all anthranilic diamide (chlorantraniliprole, cyantraniliprole, and cyclaniliprole) suggests a different response for each diamide in the field-evolved strain from Puerto Rico (PR).

**Table 4:** Dose-response to cyantraniliprole and cyclaniliprole of a Susceptible strain (SUS) and a Puerto Rico’s (PR) field-collected FAW strain.

Strain	Active Ingredients	n	^d^b	SE	^a^LC_50_ (95% CI)	^a^LC_90_ (95% CI)	^b^RR_50_	^b^RR_90_	X^2^	^c^df
PR	cyantraniliprole	231	1.5	0.2	0.749 (0.56, 1.02)	5.14 (3.23, 10.08)	11	27	2.8	3
SUS		234	2.8	0.5	0.066 (0.04, 0.07)	0.191 (0.15, 0.27)	-	-	4.3	3
PR	cyclaniliprole	286	1.4	0.2	0.3718 (0.17, 0.66)	3.28 (1.6, 13.2)	74	213	7.9	4
SUS		287	2.7	0.3	0.0051 (0.004, 0.005)	0.0154 (0.012, 0.02)	-	-	6.9	4

^a^ LC_50_ or LC_90_ (μg/cm^2^)

^b^ Resistance ratio (RR), LC_50_ of resistant strain / LC_50_ of susceptible strain or LC_90_ of resistant strain / LC_90_ of susceptible strain

^c^ df = degrees of freedom

^d^ slope

## Discussion

In this study, we characterized the inheritance and the possibility for metabolism in field-evolved resistance in a FAW population from Puerto Rico (PR) to both chlorantraniliprole (96-fold) and flubendiamide (2,762-fold) using diet overlay bioassays which probably estimate more accurate the toxicity of diamide compounds to FAW than laboratory topical bioassay [43]. Field-evolved resistance to diamides (flubendiamide and chlorantraniliprole) was first reported in 2017 using topical bioassays [15]; however, the FAW population in our study was collected three years later after 12 cropping seasons subjected to diamide insecticide selection. Since 2017 flubendiamide is no longer used to manage FAW in Puerto Rico; however, the resistance levels are still very high likely due to prior intense selection pressure by flubendiamide and cross-resistance by using chlorantraniliprole in each cropping season. Chlorantraniliprole is still used to manage FAWs in corn, despite recent field observations that efficacy has decreased (i.e. practical resistance) [44]. FAWs from Puerto Rico also exhibited cross-resistance to other diamides as we found in this study.

Cross-resistance in this case is defined as resistance to compounds of the same chemical family never used before [45]. In our unique FAW population from Puerto Rico, we observed cross-resistance to cyantraniliprole (11-fold) and high levels of cross-resistance to cyclaniliprole (74-fold), suggesting the possibility of cross-resistance development to diamides. This is particularly concerning in regions where more than two diamide compounds are deployed to manage FAW, as occurred in Asia with the recent FAW infestations [46]. Resistance and cross-resistance to four diamide compounds suggested an overlapping of the binding sites of RyRs [47]. We determined that there are high levels of field-evolved resistance of FAW from Puerto Rico without further selection in the laboratory. Similarly, resistance to chlorantraniliprole was reported in Brazil by using an F_2_ screening method in overlay diet assays from field populations as well as cross-resistance to flubendiamide (42,000-fold) and cyantraniliprole (26-fold) [33]. Resistance to these insecticides has been reported in other pests including tomato leafminer (*Tuta absoluta*) in Greece, Brazil, and Spain [48, 49], in South Korea, to beet armyworm (*Spodoptera exigua*) and diamondback moth (*Plutella xylostella*) [50, 51], in China to the Asiatic rice borer (*Chilo suppressalis*) [52] and lately reported field-evolved resistance in China to cotton bollworm (*Helicoverpa armigera*) [53].

Our inheritance of resistance analysis suggests that resistance to chlorantraniliprole in FAW from Puerto Rico is autosomal. Similar results were documented in the continental area of South America [33]. Inheritance of resistance by autosomal traits in FAW from continental areas of the Americas was previously reported to other classes of insecticides: carbamates (carbaryl) [54] and organophosphate (chlorpyrifos) [55], pyrethroids (lambda-cyhalothrin) [56], nicotinic acetylcholine receptor (nAChR) allosteric modulators, spinosyns (spinosad and spinetoram) [57, 58], glutamate-gated chloride channel (GluCl) allosteric modulators, avermectins and milbemycins (emamectin benzoate) [34] and inhibitors of chitin biosynthesis, benzoylureas (novaluron and teflubenzuron) [59, 60].

In contrast to chlorantraniliprole, flubendiamide reciprocal crosses indicates a paternal sex-linked inheritance resistance, that the resistance is conferred by the males of the parent trait. Few records of this type of inheritance have been published to date. For example, in 2016, a field-evolved resistant colony of the convergent lady beetle (*Hippodamia convergens*) (Coleoptera: Coccinellidae) from Georgia, USA presented a recessive inheritance with maternal sex-linkage in to the pyrethroid lambda-cyhalothrin [61]. Furthermore, the PR strain presented incompletely recessive resistance from the PR female strain to both chlorantraniliprole and flubendiamide. Similar results were reported in other Lepidoptera species as diamondback moth [62, 63], oriental tea tortrix (*Homona magnanima*) [64] and tomato leafminer [48]; however, the reciprocal crosses demonstrated an incompletely dominant trait from the parental PR strain.

In our reciprocal crosses, we observed that individuals, presumed to be heterozygous, exhibited incompletely recessivity (Fig 1). A factor that might skew this result is the degree of homozygous resistance of the resistance population because the strain might be a mix of homozygous resistant and heterozygous individuals that carry genes for resistance. However, the resistance levels of FAW to both diamide compounds were very high and there was little overlapping with the susceptible strain to most of the concentrations (Fig 1). Another possibility that might affect the level of heterozygosity is the presence of a lethal gene [65]. Dexter [65] noticed that in a specific stock of *Drosophila*, offspring from half of the females displayed a distinct pattern of twice as many females as males. This observation was attributed to a gene present in one of the sex chromosomes of these females that inhibits the development of any male inheriting it. We did not observe any drastic fitness cost or skew in sex in our resistant strain meaning the low probability of a lethal gene in our resistant strain. We acknowledge the limitations and assumptions of our analysis, particularly the assumption of homozygosity for resistance in the Puerto Rican (PR) population. The PR specimens could indeed be a mix of homozygotes and heterozygotes, or the resistance could be entirely dominant but also recessive lethal [66]. These scenarios are less likely, but potentially influenced the observed results of partial dominance or partial recessivity in the hybrid crosses, thereby adding a layer of complexity to the inheritance of resistance. This underlines the necessity for further research to fully understand the genetic dynamics at play. For instance, recent studies have shown that when comparing strains with genetically distant backgrounds, there could be an overestimation of the fitness cost. This might create a misleading impression that resistance carries a significant cost, and, as a result, its frequency would naturally diminish in the absence of the insecticide [67].

Despite these complexities, it remains vital to note that individuals carrying a single copy of the resistant allele might survive under conditions of decreased pesticide residue, potentially resulting in increased mutation rates of resistance genes [68] and that might result in the survival of heterozygotes when chlorantraniliprole residues breakdown days after foliar applications or after many days of the emergence of corn plants that come from diamide treated seed. This is a likely scenario, since FAW moths lay egg masses continuously during the season [69]. Therefore, to manage resistance effectively, it is essential that diamide treatments, whether seed treatment or foliar sprays, should not be repeatedly applied within the same season.

Resistance of lepidopteran pests to diamides has been considered mainly through target-site mutations that cause high levels of resistance in field-evolved and lab-selected strains [6]. Since ryanodine receptors (RyRs) are determined by a single gene [70] in *Drosophila*, target-site mutations are expected to occur due to the high selectivity on these receptors. For instance, in diamondback moth, rice stem borer (*Chilo suppressalis*), and tomato leafminer, the same polymorphism is reported (G4946E) located close to the C-terminal of the RyRs gene [6, 51, 71, 72], while in the Noctuidae family just one polymorphism has been reported (I4790M) in beet armyworm and FAW [73, 74]. In addition, the frequency of these known mutations has been studied in tomato leafminer and confirmed by CRISPR/Cas9 modification in *Drosophila*, suggesting that the target site mutations confer modifications on the action site of different diamides overlapping at the binding site of the RyRs receptors [75]. However, recent genotyping sequencing for different target-site mutations in Puerto Rico FAW samples showed the absence of the polymorphisms G4946E and I4790M [76] suggesting either different polymorphisms or a different mechanism of resistance.

In our PR strains, resistance to chlorantraniliprole is not strongly associated with detoxification enzymes including P450s, esterases, ABC transporters, as reported with for FAW in other studies [73, 77]. This result is supported from similar evidence in other Lepidoptera pests including diamondback moth [78], beet armyworm [50], and oblique-banded leafroller (*Choristoneura rosaceana*) [79]. Our findings using synergists with flubendiamide showed a moderate role of esterases and glutathione S-transferases and some involvement of ABC transporters in the detoxification process. The resistance mechanisms involved in the downregulation of ABC transporters have been observed in a field-evolved strain of diamondback moth, leading to resistance to specific molecules. Therefore, synergism of verapamil might be the first report of the role of ABC transporters on the resistance of flubendiamide in FAW. ABC transporters mutations have been recorded for resistance to the Bt proteins, Cry1F, in lab colony from Puerto Rico [80, 81]. Also, VER (p-glycoprotein inhibitor) has been used to characterize the involvement of ABC transporters in the resistance of *Rhipicephalus* (Boophilus) to pyrethroids (ivermectin) [82] and *Anopheles gambiae* to pyrethroids (deltamethrin) [83]. Further molecular analysis remains to be performed to understand this particular strain’s resistance mechanism. This is critical due to the ongoing migration pathway of the FAW, that has shown a great adaptation to those developing countries where factors such as well-established IRM programs, economic resources to acquire novel active ingredients, and efficient application methods and techniques are current limitation [84].

Puerto Rico is considered one of the most important areas around the globe for agricultural research focused on plant breeding [17, 85] due to its favorable all year around weather which allows for continuous farming. Furthermore, the identical seed regulatory frameworks in place in the U.S. and the biotechnology science-friendly environment enable a seamless exchange of seeds and uninterrupted experimentation [13]. However, favorable conditions for the crops are similar for a high pest pressure resulting in intense use of synthetic pest management tools and resistance development. This intense selection has led to resistance evolution in FAW for multiple insecticides [15]. To strengthen IPM and IRM approaches, IRAC-US and PRABIA have established an area-wide resistance management program with five critical workstreams: 1. Field efficacy trials with a different mode of actions on FAW; 2. Development and maintenance of the area-wide rotation program; 3. Scouting practices, treatments thresholds, and training; 4. Implementation and communication, and 5. Resistance monitoring bioassays [86]. Our work on the basic aspects of diamide resistance supports these workstreams and will provide the basic knowledge required for better IPM practices for these critical insect control tools. Additionally, in conjunction with the seed industry, workshops have been conducted with the research and development teams of large and medium seed companies with the same resistance problem in common research fields [86]. Restoring FAW susceptibility and enhancing the area-wide resistance management program might take time; however, these are the initial steps in the right direction to address the problem.

## Conclusion

FAW from Puerto Rico had developed field-evolved resistance to diamides. Weather and pattern of insecticide use might be similar in other areas of the world where FAW had invaded. The incomplete recessiveness of chlorantraniliprole suggests that rapid resistance might occur in other areas of the world if FAW management is followed by treatments of ryanodine receptors compounds. Also, cross-resistance to siblings’ molecules is likely to happen if IPM tools are not implement effectively. For instance, diamide seed treatment followed by diamide foliar sprays, or continuous sprays of diamide treatment. Therefore, it is critical to consolidate effective and long-term IPM programs that would prolong the active seed programs in island-type conditions of Puerto Rico that will ensure the current and near-future demand for food around the world. Lessons learned from field-evolved resistance of FAW to diamides in Puerto Rico have applications in other continents, including Africa, Asia, and Oceania.

## Supporting information

S1 Table Insecticide concentrations used in the bioassays. (XLSX)
